# Myelosuppression grading of chemotherapies for hematologic malignancies to facilitate communication between medical and dental staff: lessons from two cases experienced odontogenic septicemia

**DOI:** 10.1186/1472-6831-13-41

**Published:** 2013-08-19

**Authors:** Masaya Akashi, Yasuyuki Shibuya, Junya Kusumoto, Shungo Furudoi, Yumiko Inui, Kimikazu Yakushijin, Atsuo Okamura, Hiroshi Matsuoka, Takahide Komori

**Affiliations:** 1 Department of Oral and Maxillofacial Surgery, Kobe University Graduate School of Medicine, Kobe, Japan; 2 Division of Medical Oncology/Hematology, Department of Medicine, Kobe University Graduate School of Medicine, Kobe, Japan

**Keywords:** Hematologic malignancy, Chemotherapy, Tooth extraction, Myelosuppression grading, Odontogenic septicemia

## Abstract

**Background:**

Odontogenic diseases can be a risk factor for life-threatening infection in patients with hematologic malignancies during chemotherapy that induces myelosuppression of variable severity. Previous studies noted the necessity of the elimination of all odontogenic foci before hematopoietic stem cell transplantation. To enable planning for the adequate dental intervention, the oral medicine team must understand the general status of patient and the intensity of the chemotherapy, which is sometimes difficult to be fully appreciated by dental staff. Therefore, a simplified grading would facilitate the sharing of information between hematologists, dentists and oral hygienists. This study aimed to introduce our myelosuppression grading of chemotherapies for hematologic malignancies and analyze the timing of occurrence of severe odontogenic infection.

**Methods:**

37 patients having received various chemotherapies for hematologic malignancies were enrolled. The chemotherapy regimens were classified into four grades based on the persistency of myelosuppression induced by chemotherapy. Mild myelosuppressive chemotherapies were classified as grade A, moderate ones as grade B, severe ones as grade C, and chemotherapies that caused severe myelosuppression and persistent immunodeficiency (known as conditioning regimens for transplant) as grade D. The timing of occurrence of severe odontogenic infection was retrospectively investigated.

**Results:**

Two patients (5.4%) had severe odontogenic infections after grade B or C chemotherapy. One occurred after extraction of non-salvageable teeth; the other resulted from advanced periodontitis in a tooth that could not be extracted because of thrombocytopenia. Both were *de novo* hematologic malignancy patients. During grade D chemotherapy, no patients had severe odontogenic infections.

**Conclusions:**

The simplified grading introduced in this study is considered a useful tool for understanding the myelosuppressive state caused by chemotherapy and facilitating communication between medical and dental staff. During the period around the primary chemotherapy, especially for *de novo* hematologic malignancy patients who often received grade B to C myelosuppression chemotherapy, caution should be exercised for severe odontogenic infection by the oral medicine team, irrespective of whether invasive treatment is to be performed.

## Background

Chemotherapy and hematopoietic stem cell transplantation (HSCT), as the treatment for hematologic malignancy, result in myelosuppression and increase the susceptibility of patients to severe infections. Dentists and oral hygienists have important roles in managing the oral health of these patients [1,2]. Although it is recommended that all odontogenic foci that are potential sources of systemic infection be eliminated by the appropriate prophylactic dental treatment prior to initiation of chemotherapy [3-7], the extraction of suspected infected teeth is controversial because abnormal bleeding, insufficient wound healing and local infection can delay medical treatment for the hematological disease [8-10]. Therefore, invasive dental treatment should be carefully planned and executed at the appropriate phase. To enable planning for the invasive treatments (e.g. dental extraction and subgingival scaling), the oral medicine team must understand the general status of patient, the intensity of the chemotherapy, and the schedule for future treatment. Because it is sometimes difficult for dental staff to appreciate fully the myelosuppressive intensity of various chemotherapy regimens, a simplified grading would facilitate the sharing of information between hematologists, dentists and oral hygienists. We attempted to establish at our hospital a system of grading chemotherapy by its myelosuppressive intensity to allow the sharing of appropriate information between medical and dental staff.

This retrospective study had two purposes, one of which was to introduce our myelosuppression grading of chemotherapies for hematologic malignancies. The other was to investigate the timing of occurrence of severe odontogenic infection, because few studies determined the correlation between the myelosuppresive intensity of chemotherapy and the occurrence of sever odontogenic infection. We experienced two patients having odontogenic septicemia during chemotherapeutic treatment and analyzed the timing of those occurrences.

## Methods

A retrospective study was carried out in 37 patients having received treatments for hematologic malignancies. From January 2009 through December 2010, patients were referred from the Division of Medical Oncology/Hematology to the Department of Oral and Maxillofacial Surgery at the Kobe University Hospital to be screened for odontogenic foci.

When the first dental screening was performed, patient details, including age, gender and diagnosis were obtained. At the same time, the dental staff was informed by the hematologists of the intensity of the scheduled chemotherapy, based on the myelosuppression grading. The myelosuppressive intensity of grade A chemotherapies included the following oral agents and infusions: tyrosine kinase inhibitors for chronic myeloid leukemia; all-trans retinoic acid (ATRA) for acute promyelocytic leukemia; fludarabine phosphate internal use or intravenous drip, rituximab monotherapy, and etoposide internal use for chronic lymphoid leukemia or malignant lymphoma; melphalan plus prednisone (MP) for multiple myeloma, etc. These chemotherapies were mostly performed for outpatients and the myelosuppressive intensity was mild. The myelosuppressive intensity of grade B chemotherapies that included many different regimens (e.g. consolidation regimens for leukemia; CHOP, ABVD and ESHAP for malignant lymphoma) was moderate. The myelosuppressive intensity of grade C chemotherapies that included remission induction therapy for acute leukemia was severe. The chemotherapies that caused the most severe myelosuppression and persistent immunodeficiency (known as myeloablative conditioning regimens) were classified as grade D. The chemotherapy regimens encountered in this study are shown in Table 1.

**Table 1:** Myelosuppression grading of chemotherapies for hematologic malignancies

**Myelosuppression grading**	
Grade A	mild myelosuppression
Grade B	moderate myelosuppression (2–3 weeks for bone marrow recovery)
Grade C	severe myelosuppression (4 weeks for bone marrow recovery)
Grade D	severe myelosuppression and persistent immunodeficiency
Regimens	
Grade B	*Consolidation therapy for leukemia*:
	DA (DNR, Ara-C); MA (MIT, Ara-C); high-dose Ara-C
	*Chemotherapy for Malignant lymphoma*:
	ABVD (ADR, BLM, VLB, DTIC); CHOP (CPA, ADR, VCR, PSL); ESHAP (ETP, Ara-C, CDDP, mPSL); Hyper-CVAD/MA (course 1: CPA, VCR, ADR, DEX, course 2: MTX, Ara-C, mPSL)
Grade C	*Remission induction therapy for acute leukemia*:
	ATRA, IDR, Ara-C; DCM (DNR, Ara-C, 6-MP); DNR, VCR, CPA, L-Asp, PSL; HAM (high dose Ara-C, MIT); IDR, Ara-C
	*High****-****dose chemotherapy with peripheral blood stem cell harvest*:
	high dose VP-16
	*Salvage chemotherapy for T-cell lymphoma*:
	SMILE (MTX, ETP, IFM, L-Asp, DEX)
Grade D	*Conditioning regimen for transplant*:
	MCVC (MCNU, CBDCA, ETP, CPA); HD-ICE (IFM, CBCDA, VP-16); Flu/BU; Flu/Mel/TBI; TBI/CY

*ADR* adriamycin, *Ara-C* cytarabine, *BLM* bleomycin, *BU* Busulfan, *CBDCA* carboplatin, *CDDP* cisplatin, *CPA/CY* cyclophosphamide, *DEX* dexamethasone, *DNR* daunorubicin, *DTIC* dacarbazine, *ETP/VP-16* etoposide, *Flu* fludarabine, *IDR* idarubicin, *IFM* ifosfamide, *L-Asp* L-asparaginase, *MCNU* ranimustine, *Mel* melphalan, *MIT* mitoxantrone, *mPSL* methylprednisolone, *MTX* methotrexate, *PSL* prednisolone, *TBI* total body irradiation, *VCR* vincristine, *VLB* vinblastine, *6-MP* 6-mercaptopurine.

The dental status of all patients was evaluated by two dentists (a resident of the postgraduate program and a senior dentist in Oral Surgery). Screening consisted of clinical examination of the hard and soft oral tissues and radiographic survey, including panoramic and occasional periapical films for symptomatic teeth. All dental complications during treatment for hematologic malignancies were recorded for each patient, including infections, gingivitis, caries, pulpitis, apical periodontitis, marginal periodontitis, and pericoronitis of the third molar. Dental foci that caused infections during the period of immunocompromise were defined as apical and marginal periodontitis, and symptomatic third molar. Additionally, a complete blood count (hemoglobin, hematocrit, white blood cells, platelets, etc.) was performed to avoid the risk of infections and hemorrhage and to determine coagulation status. After confirming that the patient could tolerate invasive procedures, all symptomatic third molars and non-salvageable teeth with advanced marginal or apical periodontal disease were extracted with prophylactic antibiotic coverage to protect against oral and generalized infections.

Any patients with local signs and symptoms consistent with odontogenic infections (e.g. gingival swelling and/or dental pain) were given a dental examination and treated as necessary. The occurrence of minor (e.g. gingivitis, minor gingival bleeding, and toothache) or severe (e.g. cellulitis and sepsis) odontogenic infections was recorded and monitored throughout the chemotherapy. Detailed clinical courses of severe odontogenic infections were retrospectively analyzed.

This study was approved by the Medical Ethics Committee of Kobe University. This retrospective analysis was completed within the guidelines of the Helsinki declaration.

## Results

Patient characteristics are summarized in Table 2. Medical treatment for hematologic malignancy was transplant in 14 cases and chemotherapy alone in 23. The source of stem cell was bone marrow in seven cases, peripheral blood in five, and cord blood in two. Autologous transplant was performed in four cases, whereas the remaining ten patients received allogeneic transplant. Grade B chemotherapies (moderate myelosuppression) were performed in 34 patients (91.9%), grade C (severe myelosuppression) in 18 (48.6%), and grade D (severe myelosuppression and persistent immunodeficiency) in 15 (40.5%). Some patients were graded several times. Total courses of grade B chemotherapy were 64, grade C 22, and grade D 15.

**Table 2:** Patient characteristics

	**Number of patients (n = 37) (%)**
Median age (range)	50 (23–70)
Gender	
Male	23 (62.2)
Female	14 (37.8)
Diagnosis	
Acute myeloid leukemia	9 (24.3)
Acute lymphocytic leukemia	3 (8.1)
Acute biphenotypic leukemia	1 (2.7)
Malignant lymphoma	20 (54.1)
ATLL	3 (8.1)
Myelodysplastic syndrome	1 (2.7)
Medical treatment	
Allogeneic BMT	7 (18.9)
Autologous PBSCT	4 (10.8)
Allogeneic PBSCT	1 (2.7)
CBT	2 (5.4)
Chemotherapy alone	23 (62.2)
Myelosuppression grading [courses]*	
Grade B	34 (91.9) [64]
Grade C	18 (48.6) [22]
Grade D	15 (40.5) [15]

*ATLL* Adult T-cell leukemia/lymphoma, *BMT* Bone marrow transplantation, *PBSCT* Peripheral blood stem cell transplantation.

*CBT* Cord blood transplantation.

*Course of treatment was counted. Some patients were graded several times.

The extraction of 45 non-salvageable teeth from 10 patients was performed following administration of prophylactic antibiotics. On the day of operation, the median white blood cell (WBC) count was 4100/μl (range: 1300–11300) and the platelet count was 22.8 × 10^4^/μl (range: 2.4-43.1) (Table 3). Platelet transfusion was required in one patient.

**Table 3:** Degree of myelosuppression at the point of dental extraction

Number of removed teeth	48
Number of patients*	10
WBC (/μl), median (range)	4100
Plt (×10^4^/μl), median (range)	22.8

*Extraction was performed multiple times in five patients.

*WBC* white blood cell count.

*Plt* platelet count.

Although all patients received the scheduled chemotherapy with no alteration, interruption or delay caused by the dental treatment, in two of the 37 patients (5.4%), severe odontogenic infections occurred. These cases are discussed in detail below.

### Case 1

A 57-year-old female with B-cell lymphoma received CHOP therapy (myelosuppression grade B) on the day of hospitalization. The primary dental examination was performed 10 days after the start of chemotherapy. When bone marrow recovered on the 19th day, non-salvageable teeth with marginal periodontitis were extracted in preparation for subsequent autologous peripheral blood stem cell transplantation.

**Figure 1:**
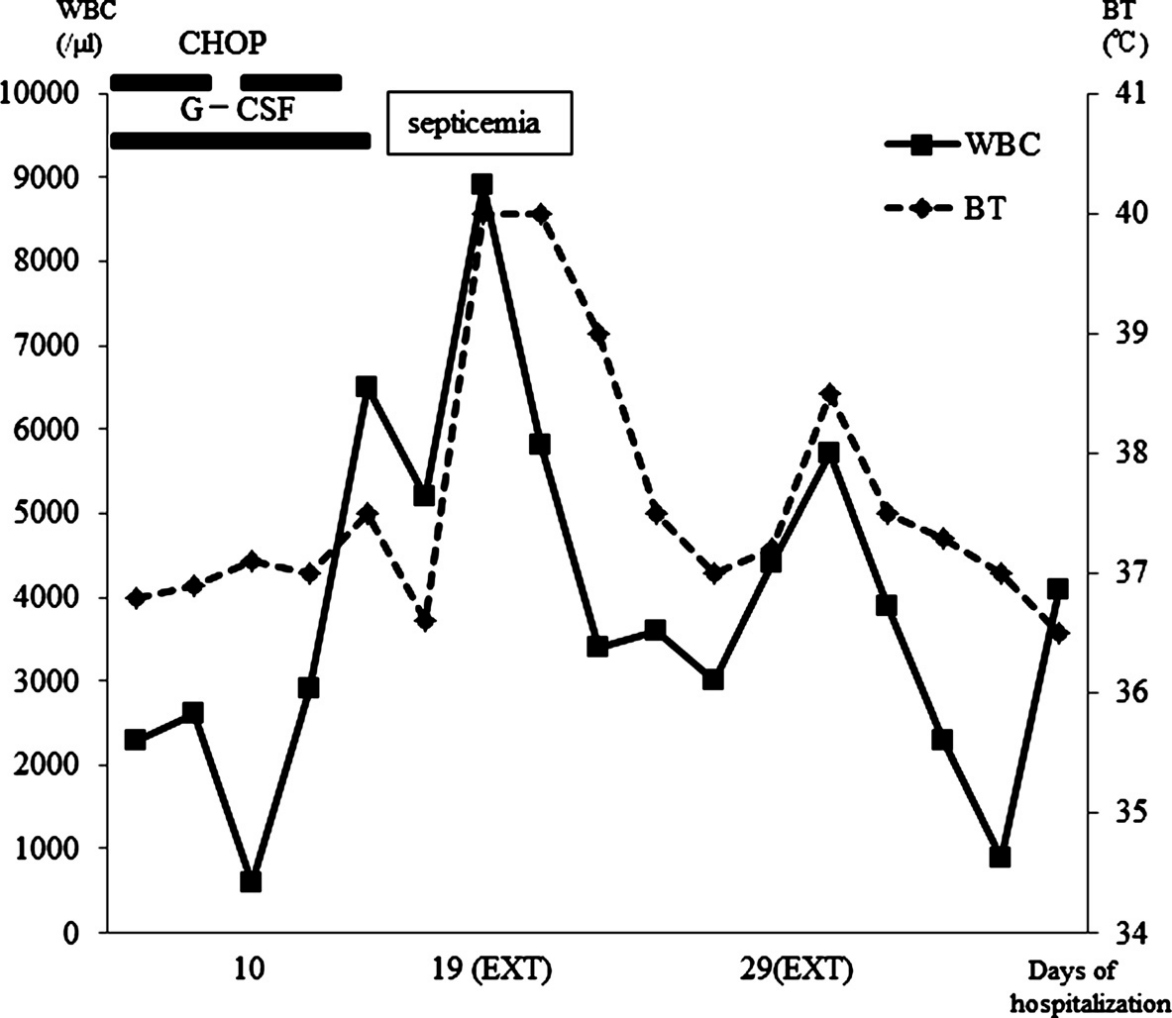
**Summary of case 1.** G-CSF, granulocyte colony stimulating factor; BT, body temperature; EXT, tooth extraction; WBC, white blood cells.

During the night after the procedure, body temperature became elevated (Figure 1). Based on clinical findings and laboratory data, the patient was diagnosed as having sepsis with disseminated intravascular coagulation resulting from infection after dental extraction. She received intravenous antibiotics (meropenem, clindamycin and teicoplanin). The patient duly recovered, and the remaining non-salvageable teeth were extracted 10 days after the onset of septicemia.

### Case 2

A 67-year-old male with myelodysplastic syndrome-derived overt leukemia received remission induction therapy (IDA, Ara-C) on the day of hospitalization. He underwent primary dental examination 5 days after the start of grade C myelosuppression chemotherapy. Whereas some asymptomatic teeth with advanced marginal periodontitis were observed, invasive treatments (i.e. tooth extraction) could not be provided because of thrombocytopenia caused by hematologic tumor and chemotherapy (platelet count was 2.1 × 10^4^/μl). Dental treatment was restricted to conservative therapy such as oral hygiene instruction.

**Figure 2:**
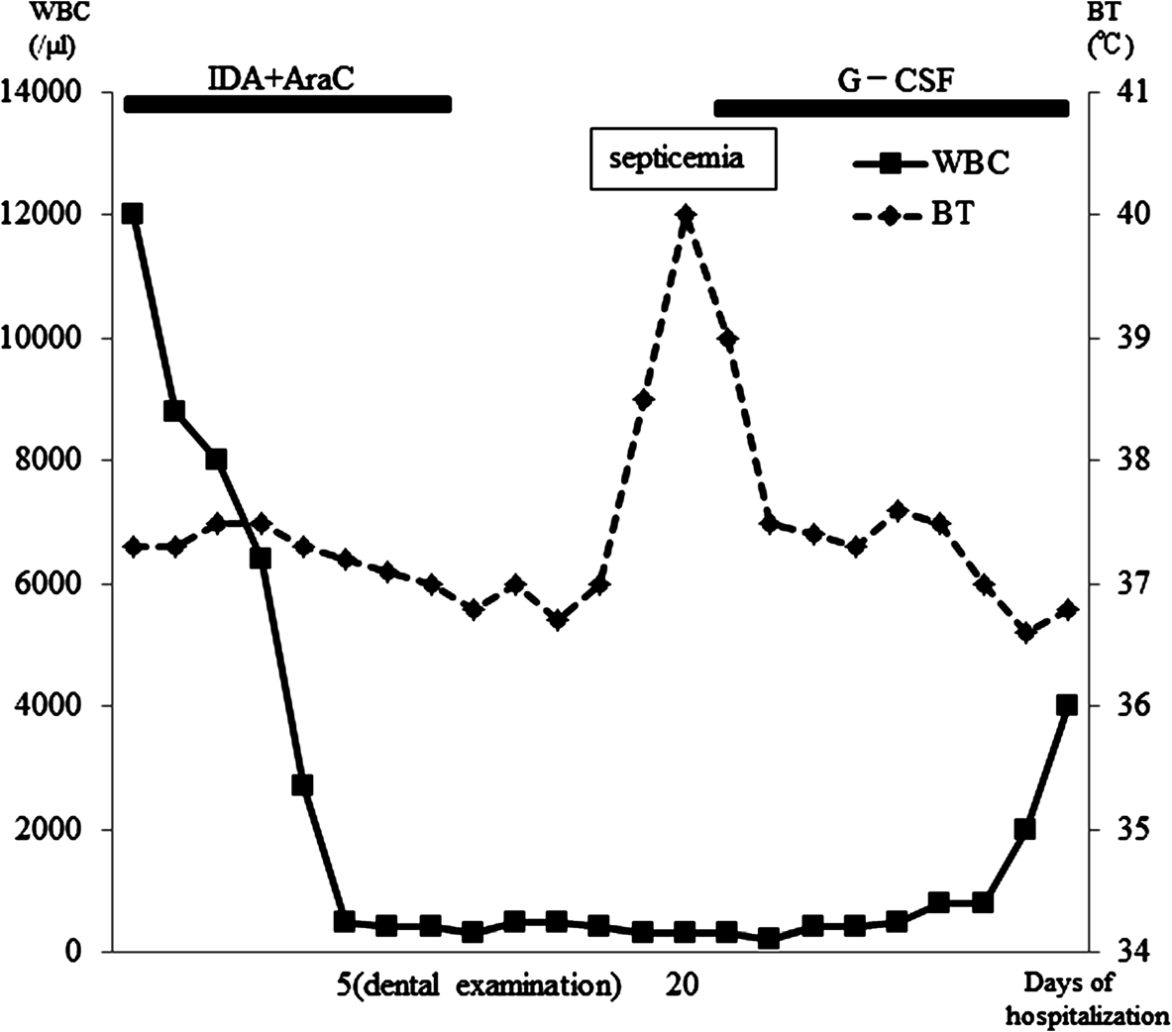
**Summary of case 2.** G-CSF, granulocyte colony stimulating factor; BT, body temperature; EXT, tooth extraction; WBC, white blood cells.

When the WBC count dropped to 400/μl during the 20 days after initiation of chemotherapy, body temperature rose to 39°C (Figure 2). Pulse rate was over 100 beats per minute and respiratory rate was over 20 breaths per minute. The clinical examination revealed severe gum pain and broad swelling around a tooth with advanced marginal periodontitis. Although the results of bacterial detection (2 trials of blood culture) were negative, clinical findings indicated the sepsis syndrome resulting from odontogenic infection [11]. The infection was resolved by administration of antibiotic drugs (cefepime, hydrochloride and clindamycin).

## Discussion

In our hospital, hematologists attempted to grade the myelosuppressive properties of each chemotherapy regimen to facilitate effective communication with the oral medicine team. Although dentists and oral hygienists must understand the general status of patient and the intensity of the chemotherapy to enable planning for the adequate dental intervantion, it is sometimes difficult for dental staff to appreciate fully the myelosuppressive intensity of the chemotherapy regimens. The simplified grading introduced in this study is considered a useful tool for understanding the myelosuppressive state caused by chemotherapy and facilitating communication between medical and dental staff. Based on this grading, we found that two patients out of 37 suffered from sepsis resulting from odontogenic diseases and investigated the timing of those occurrences.

In case 1, the sepsis happened after the grade B chemotherapy. The patient received granulocyte colony-stimulating factor (G-CSF) because granulocytopenia occurred (the nadir of the WBC count was 600/μl). Whereas laboratory data improved, the patient experienced sepsis following invasive dental treatment 19 days after the start of the first course of CHOP. Owing to the chemotherapy and residual tumor, this patient may not have recovered full immune function, despite having apparently normal WBC count. In case 2, the patient suffered sepsis after grade C chemotherapy. This chemotherapy was initiated very rapidly after admission because tumor growth was quite rapid. Although professional dental care had not be performed before hospitalization and his dental hygiene was so poor on primary dental examination, the delay in initiating chemotherapy that would have been caused by pursuing dental treatment would have been unacceptable. We noted that both patients were *de novo* hematologic malignancy patients that were sick, febrile and hemorrhagic owing to massive tumor volume, and were thus in a myelodeficient state. Despite their illness, primary dental examination was important, given that previous reports have suggested that prophylactic dental treatment is a critical factor in reducing the occurrence of infections during chemotherapy [12]. The time available for providing prophylactic dental treatment influences the incidence of infection, but elimination of all odontogenic foci takes considerable time [13-15]. Yamagata et al. recommended that the dental extraction should be performed during remission and 10–14 days before the start of conditioning [16]. Raber-Durlacher et al. mentioned that the intervals between chemotherapy cycles may provide a good opportunity for improving oral and periodontal health [17]. During neutropenia, invasive procedures, such as periodontal probing, should be avoided.

The findings of this study may indicate that myelosuppression grade B-to-C chemotherapies may place the patient at the risky phase of experiencing severe odontogenic infections, perhaps because these types of chemotherapies are commonly given to patients with *de novo* hematologic malignancies. These patients have immunodeficiency and thrombocytopenia resulting from untreated tumor volume and chemotherapy and, as seen in the patients in this study, tend to have poor oral hygiene. Immune status in these patients is hard to judge from purely laboratory data. Thus, caution should be exercised by the oral medicine team when considering grade B to C chemotherapies especially for *de novo* hematologic malignancy patients, irrespective of whether invasive treatment is to be performed. In our study, odontogenic septicemia did not occur in 15 patients during grade D chemotherapy that had caused severe immunosuppression and persistent immunodeficiency. It is clear that reduction of tumor volume by grade B-to-C chemotherapy (known as induction or consolidation chemotherapy) can be safely followed by HSCT therapy, provided that adequate prophylactic dental treatments during the intervals between chemotherapy cycles. This hypothesis may be supported by one previous important case report by Soga et al. [18]. In their report, the frequency of febrile neutropenia decreased with increasing cycles of chemotherapy, and decreases in febrile neutropenia corresponded to the progress of periodontal treatment. The dental interventions performed could have decreased the occurrence of exacerbations of dental infections in a subsequent phase, which may explain why such exacerbations were not observed during HSCT.

The period around primary chemotherapy for *de novo* hematologic malignancy patients is considered to be a risky phase with regard to the development of severe odontogenic infections owing to instability in the immune system caused by the myelosuppressive chemotherapy and the untreated hematologic tumor. The oral medicine team should be mindful of rectifying poor oral hygiene to negate this risk with conservative therapies, but dentists should avoid radical treatment during this period. When the status of *de novo* hematologic malignancy patients is improved by grade B or C chemotherapy, invasive procedures should be performed rapidly during the intervals between chemotherapy cycles, and completed before the initiation of grade D chemotherapy.

A standard dental management protocol for use in patients prior to chemotherapy would be useful adjunct to treatment planning. Yamagata et al. have previously described detailed criteria for minimal intervention treatment of detrimental dental disorders before HSCT [19]. A prospective study is required to evaluate the effectiveness of a newly designed dental treatment protocol combined with myelosuppression grading of chemotherapy. The main issues of this study were the small sample size, the heterogeneity of study population with respect to diagnosis as well as type of treatment, and the lack of direct bacterial evidence from blood culture results. Oral mucositis that is a common complication associated with high-dose chemotherapy and can be a portal of entry for systemic infection [20] as well as odontogenic disease could not be evaluated in this study. The recent study revealed that the dental/periodontal infections can be easily overlooked or misdiagnosed, particularly when symptoms on inflammation are masked by neutropenia [21]. Therefore, the frequency of dental/periodontal infections is likely underestimated. More studies are needed on the relative contributions of odontogenic infections to local and systemic complications during the myelosuppressive chemotherapy. The use of myelosuppression grading to aid in structuring prophylactic dental treatment has great potential in reducing oral complications in patients with hematologic malignancies, but requires further validations.

## Conclusion

The simplified grading introduced in this study is considered a useful tool for understanding the myelosuppressive state caused by chemotherapy and facilitating communication between medical and dental staff. During the period around the primary chemotherapy for *de novo* hematologic malignancy patients, caution should be exercised for the occurrence of severe odontogenic infection due to immunodeficiency resulting from untreated tumor and poor oral hygiene.

## Competing interests

The authors declare that they have no competing interest.

## Authors’ contributions

MA designed the study, performed the data analyses, and drafted the manuscript. YS contributed to study design and data analysis. SF, YI, KY and AO contributed to data collection and analysis. JK collected data. HM and TK revised the article for important intellectual content. All authors approved the final manuscript.

## Pre-publication history

The pre-publication history for this paper can be accessed here:

http://www.biomedcentral.com/1472-6831/13/41/prepub
